# Joint Microbial and Metabolomic Network Estimation with the Censored Gaussian Graphical Model

**DOI:** 10.1007/s12561-020-09294-z

**Published:** 2020-09-21

**Authors:** Jing Ma

**Affiliations:** Division of Public Health Sciences, Fred Hutchinson Cancer Research Center, Seattle, WA 98109 USA

**Keywords:** Data integration, Microbiome, Metabolomics, Censored Gaussian graphical models, Conditional dependence

## Abstract

Joint analysis of microbiome and metabolomic data represents an imperative objective as the field moves beyond basic microbiome association studies and turns towards mechanistic and translational investigations. We present a censored Gaussian graphical model framework, where the metabolomic data are treated as continuous and the microbiome data as censored at zero, to identify direct interactions (defined as conditional dependence relationships) between microbial species and metabolites. Simulated examples show that our method metaMint performs favorably compared to the existing ones. metaMint also provides interpretable microbe-metabolite interactions when applied to a bacterial vaginosis data set. R implementation of metaMint is available on GitHub.

## Introduction

The field of microbiome research is shifting rapidly from cataloging the taxonomic compositions of microbial communities [1] to refined technologies that capture strain-level variations or amplicon sequence variants [2–4] and to multi-omics studies that better capture community functional activity [5]. In particular, metabolomics has been extremely useful in explaining microbial functional potential because of its capability in tracking microbially derived metabolites [6–8]. Associations between specific microbes and metabolites provide key insights and improved mechanistic models of host-microbe interactions [9–12]. In practice, the non-parametric Spearman’s rank correlation is often used to quantify the pairwise correlation between microbes and metabolites. However, Spearman’s rank correlation only captures marginal monotonic association and does not distinguish direct and indirect interactions. In contrast, partial correlations measure conditional dependencies and allow the identification of direct interactions between microbes and metabolites [13].

One analytical challenge specific to the microbiome data are the uneven sequencing depths that arise due to differential efficiency of the sequencing process. The total number of reads in a sample is also constrained by the biological specimen at hand and does not reflect the absolute abundance present in the ecosystem. A common practice to address this issue is to transform the raw counts into relative abundances by normalizing over the total sequencing reads in each sample. In other words, raw sequencing counts are transformed into proportions of different microbes whose sum has to be one, also known as compositional data. Several lines of work have been proposed to model marginal and/or conditional microbial interactions from compositional data. For example, SparCC [14] and CCLasso [15] both estimate the linear Pearson correlations between log-transformed counts. A major limitation of marginal association measures such as the Pearson correlation is that they cannot distinguish between direct and indirect relationships [16]. To address this issue, SPIEC-EASI [17] learns the conditional dependencies between pairs of microbes while adjusting for effects from other species in the analysis. This is achieved by estimating the inverse covariance of the centered log-ratio (clr) transformed data using e.g., the graphical lasso algorithm [18]. Fang et al. [19] assume that the observed relative abundances follow the logistic normal distribution and proposed a Majorization-Minimization algorithm for learning the conditional dependence relationships among microbes.

**Fig. 1:**
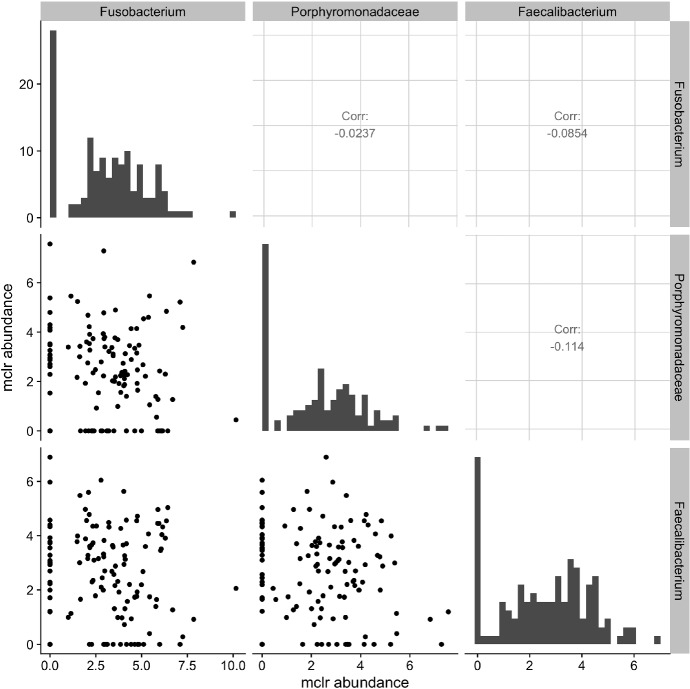
Scatter plots of the modified centered log-ratio (mclr) transformed abundances of 3 bacterial species in the vaginal microbiome data from McMillan et al. [9]. Marginal distribution of each species is illustrated along the diagonal. The upper panels show the Pearson correlations between pairs of species

Many of the aforementioned methods are specific to microbiome data and are not directly applicable for joint analysis of microbiome and other omics data types. One naive approach for joint estimation is to apply the graphical lasso algorithm directly to clr transformed microbiome and metabolomic data. However, as illustrated in Fig. 1, the Gaussian graphical model may be a poor fit for microbiome data because the marginal distributions of transformed raw counts are in fact highly skewed and often zero inflated.

This motivates the need for new statistical methodology that can accommodate both microbiome and metabolomic data while accounting for the zero inflation in microbial abundance. Some zero values are sampling zeros that arise due to limited sequencing depths, whereas others are biological zeros that indicate complete absence of a species [20]. Silverman et al. [21] in an unpublished manuscript illustrated that biological zeros in many applications can be approximated as sampling zeros because they both represent a truly low abundance. In this paper, we treat the observed zeros as due to undersampling, and propose a censored Gaussian graphical model (cGGM) to infer the conditional dependencies among microbes and metabolites. Specifically, let ${\varvec{W}}=(W_1, \ldots , W_q)^{\intercal }$ with $W_j>0$ for all *j* be the latent variables, called *the basis*, that represent the true absolute abundance for each species. Due to undersampling and uneven sequencing depths, the observed abundance ${\varvec{R}}$ is related to ${\varvec{W}}$ via

1 $$\begin{aligned} R_j = N W_j {\varvec{I}}(\log W_j > u_j) , \end{aligned}$$

where $N>0$ is a scaling factor that may depend on ${\varvec{W}}$, $u_j$ is a constant which indicates the limit of detection for the *j*-th variable, and ${\varvec{I}}(\cdot )$ is the indicator function. The censoring value $u_j$ may be known from the experiment or estimated from data. To adjust for the uneven sequencing depths, we apply the modified clr (mclr) transformation to ${\varvec{R}}$, which transforms all non-zero counts using the usual clr and shifts all transformed values to be strictly positive [22]. The diagonal panels in Fig. 1 show the histograms of mclr transformed abundances. Compared to the usual clr transformation that requires a pseudo count when dealing with zeros, mclr preserves the ranking of observed counts across multiple samples and is less biased towards rare species [22]. Denote ${\varvec{X}}_1 = {\text{mclr}}_{\varepsilon }({\varvec{R}})$ the resulting vector after mclr transformation with parameter $\varepsilon$, which we elaborate in Sect. 2.3. Let ${\varvec{X}}_2=(X_{q+1},\ldots , X_{p})^{\intercal }$ denote the log transformed concentration measures from $p-q \ (p>q)$ metabolites. A natural model for integrating microbiome and metabolomic data is to assume that ${\varvec{X}}_1$ and ${\varvec{X}}_2$ follow a censored multivariate normal distribution with mean ${\varvec{\mu }}$ and covariance $\varSigma$. Zero entries in the inverse covariance matrix $\varOmega =\varSigma ^{-1}$ capture the conditional independence relationships among the microbes and metabolites.

The problem of inferring the joint microbe-metabolite network thus reduces to estimating $\varOmega$ from *n* independent and identically distributed observations on $({\varvec{X}}_1,{\varvec{X}}_2)$. We provide metaMint which is based on estimating each pair of marginal correlations with maximum likelihood. Given the estimated correlation matrix, metaMint uses the graphical lasso to recover the conditional dependencies between microbes and metabolites (direct interactions). We compare our method with several existing approaches in simulations, and show that metaMint outperforms the others in network structural recovery and accuracy of estimating the inverse covariance matrix. When applied to a real data on bacterial vaginosis [9], the integrated network reveals biologically relevant microbe-metabolite interactions and also identifies novel interactions that may serve as potential biomarkers for diagnosis and treatment of bacterial vaginosis.

The censored multivariate normal distribution has been commonly used to analyze environmental data that are often subject to pre-specified detection limits. For example, Hoffman and Johnson [23], Pesonen et al. [24] and Jones et al. [25] studied covariance estimation for left censored multivariate normal distribution in the classic low-dimensional setting. Recently, Augugliaro et al. [26] proposed an approximated EM algorithm for inverse covariance estimation in the high-dimensional setting and applied the method to single-cell data. The work by McDavid et al. [27] was also motivated by single-cell data, but the authors proposed the zero-inflated Gaussian graphical model, which treats zeros as coming from a degenerate point mass at zero instead of being censored. Compared to existing literature, our contribution is a unified model for joint estimation of the integrated microbe and metabolite network in the high-dimensional setting. Our algorithm works well in a variety of scenarios.

The rest of the paper is organized as follows. In Sect. 2, we describe the censored Gaussian graphical model framework and the proposed algorithm. We present extensive numerical studies in Sect. 3 and a real data example on bacterial vaginosis in Sect. 4. We conclude our paper with discussions in Sect. 5.

## The Censored Gaussian Graphical Model

The censored Gaussian graphical model is suitable for zero-inflated data, which is often the case with microbiome data as shown in Fig. 1. In practice, it is reasonable to assume that the observed zeros are due to undersampling or censoring from below.

### Definition 1

A random vector ${\varvec{X}}$ is said to follow a censored multivariate normal distribution with mean ${\varvec{\mu }}$ and covariance $\varSigma$ if there exists constants $u_1, \ldots , u_p$ such that $X_j=Y_j {\varvec{I}}(Y_j > u_j) + u_j {\varvec{I}}(Y_j \le u_j)$ where

$$\begin{aligned} {\varvec{Y}}\sim N({\varvec{\mu }}, \varSigma ) . \end{aligned}$$

The censoring values ${\varvec{u}}= (u_1,\ldots , u_p)^\intercal$ are experiment specific and can be inferred from data. For example, one can use the smallest value that occurs more than a pre-specified threshold (e.g. 10%) as an estimate. A pre-specified threshold is necessary to ensure that the smallest value occurs more often than by chance. For zero-inflated microbiome data, the censoring values are set to be 0. When there is no censoring in the *j*-th variable, we set $u_j=-\infty$.

The density of the multivariate normal distribution with mean ${\varvec{\mu }}$ and inverse covariance $\varOmega =\varSigma ^{-1}$ is

$$\begin{aligned} \phi ({\varvec{y}}; {\varvec{\mu }}, \varOmega ) = (2\pi )^{-p/2} |\varOmega | ^{1/2} \exp \left\{ ({\varvec{y}}-{\varvec{\mu }})^\intercal \varOmega ({\varvec{y}}-{\varvec{\mu }})\right\} . \end{aligned}$$

Without loss of generality, let ${\varvec{X}}= ({\varvec{X}}_o, {\varvec{X}}_c)$ where ${\varvec{X}}_o$ denotes the uncensored components and ${\varvec{X}}_c$ denotes the censored components. Given censoring values ${\varvec{u}}= (-\infty ,\ldots ,-\infty , {\varvec{u}}_c)$, the density function of ${\varvec{X}}$ is

2 $$\begin{aligned} \psi ({\varvec{x}}_o, {\varvec{u}}; {\varvec{\mu }},\varOmega ) = \int _{{\varvec{u}}_c}^{\infty } \phi ({\varvec{x}}_o,{\varvec{x}}_c; {\varvec{\mu }},\varOmega ) \text{{d}} {\varvec{x}}_c = \phi ({\varvec{x}}_o;{\varvec{\mu }},\varOmega ) \int _{{\varvec{u}}_c}^{\infty } \phi ({\varvec{x}}_c\mid {\varvec{x}}_o; {\varvec{\mu }},\varOmega ) \text{{d}} {\varvec{x}}_c. \end{aligned}$$

Let $\{{\varvec{x}}^{(1)}, \ldots , {\varvec{x}}^{(n)}\}$ denote a set of *n* independent and identically distributed observations on ${\varvec{X}}$. In high-dimensional settings, a natural strategy to estimate the inverse covariance matrix is to maximize the $\ell _1$ penalized loss function

3 $$\begin{aligned} \frac{1}{n} \sum _{i=1}^n \log \psi ({\varvec{x}}^{(i)}, {\varvec{u}}; {\varvec{\mu }}, \varOmega ) - \lambda _n \sum _{1\le j<k\le p}|\varOmega _{jk}|, \end{aligned}$$

where $\lambda _n$ is a regularization parameter that controls the sparsity of $\varOmega$. However, direct optimization of (3) is challenging due to the integral in (2) over a potentially high-dimensional space. Augugliaro et al. [26] studied a general version of (3) where variables can be left and right censored. They proposed to use the EM algorithm to optimize the expectation of the full log-likelihood with respect to the conditional distribution ${\varvec{X}}_c\mid {\varvec{X}}_o$. However, exact optimization of the EM algorithm is computationally challenging as it requires the second moment of ${\varvec{X}}_c \mid {\varvec{X}}_o$, which is a multivariate truncated Gaussian. The approximation in Augugliaro et al. [26] is adapted from Guo et al. [28] and only works well when the inverse covariance matrix is very sparse or the regularization parameter $\lambda _n$ is large.

### A Direct Estimator Via Marginal Correlations

Our proposal metaMint is based on estimating the marginal correlations directly. A similar idea was used to estimate the correlation matrix of ordinal graphical models [29], where the authors showed that the direct estimator achieves more accurate estimation of the inverse covariance matrix compared to the approximated EM approach in Guo et al. [28].

The first step in metaMint is to estimate the marginal distribution for each variable, which can be done by fitting a univariate Tobit model [30] and has been implemented in the R package censReg [31]. Let ${\hat{\mu }}_j$ and ${\hat{\sigma }}_j^2$ be, respectively, the estimate of the mean and variance for the *j*-th variable. It can be shown that ${\hat{\mu }}_j$ is a consistent estimate of $\mu _j$, and ${\hat{\sigma }}_j^2$ is consistent for $\sigma _j^2=\varSigma _{jj}$. To find the empirical covariance matrix ${\hat{\varSigma }}$, it suffices to estimate each pairwise correlation.

Suppose we have two variables $X_j$ and $X_k\ (j<k)$. If no observation is censored, it is straightforward to estimate their correlation using the Pearson’s correlation coefficient. In the following, we provide details on correlation estimation when at least one variable is censored.

Consider first the case where both variables $X_j$ and $X_k$ are censored from below with $u_j$ and $u_k$, respectively. For the *i*-th observation, let $\eta _{ij} = {\varvec{I}}(x^{(i)}_j>u_{j})$ be the indicator function of whether the *j*-th variable is censored. The pairwise joint log-likelihood can be written as a function of the correlation $\rho _{jk}$,

$$\begin{aligned} \ell ^{(i)}_1 (\rho _{jk}; \mu _j, \mu _k, \sigma _j^2, \sigma _k^2) &=\eta _{ij}\eta _{ik} \log \text {P}(Y_j = x^{(i)}_j, Y_k = x^{(i)}_k) \\&+ \eta _{ij} (1-\eta _{ik}) \log \text {P}(Y_j = x^{(i)}_j, Y_k< u_{k}) \\&+ (1-\eta _{ij}) \eta _{ik} \log \text {P}(Y_j< u_{j}, Y_k = x^{(i)}_k) \\&+ (1-\eta _{ij}) (1-\eta _{ik}) \log \text {P}(Y_j< u_{j}, Y_k < u_{k}), \end{aligned}$$

where $Y_j$ and $Y_k$ are bivariate normal with mean $(\mu _j, \mu _k)^\intercal$ and covariance

$$\begin{aligned} \begin{pmatrix} \sigma _j^2 &{} \rho _{jk} \sigma _j\sigma _k\\ \rho _{jk} \sigma _j\sigma _k &{} \sigma _k^2 \end{pmatrix}. \end{aligned}$$

Let $\phi (\cdot )$ and $\varPhi (\cdot )$ denote, respectively, the density and the cumulative distribution function (c.d.f.) of a standard normal variable. Let the c.d.f. of a bivariate standard normal variable with correlation $\rho$ be $\varPhi _2(u,v,\rho )$. The conditional distribution $Y_k \mid Y_j=x^{(i)}$ is again a normal distribution with mean ${\tilde{\mu }}_k = \mu _k + \frac{\sigma _k}{\sigma _j}\rho _{jk}(x^{(i)}_j - \mu _j)$ and standard deviation ${\tilde{\sigma }}_k = \sigma _k \sqrt{1-\rho _{jk}^2}$. The pairwise joint log-likelihood thus becomes

$$\begin{aligned} \ell _1^{(i)} (\rho _{jk}; \mu _j, \mu _k, \sigma _j^2, \sigma _k^2) =&\eta _{ij}\eta _{ik} \log \left\{ \frac{1}{{\tilde{\sigma }}_k} \phi \left( \frac{x^{(i)}_k - {\tilde{\mu }}_k}{{\tilde{\sigma }}_k} \right) \frac{1}{\sigma _j} \phi \left( \frac{x^{(i)}_j-\mu _j}{\sigma _j} \right) \right\} \\&+\eta _{ij}(1-\eta _{ik}) \log \left\{ \varPhi \left( \frac{u_{k} - {\tilde{\mu }}_k}{{\tilde{\sigma }}_k} \right) \frac{1}{\sigma _j} \phi \left( \frac{x^{(i)}_j-\mu _j}{\sigma _j} \right) \right\} \\&+(1-\eta _{ij})\eta _{ik} \log \left\{ \varPhi \left( \frac{u_{j} - {\tilde{\mu }}_j}{{\tilde{\sigma }}_j} \right) \frac{1}{\sigma _k} \phi \left( \frac{x^{(i)}_k-\mu _k}{\sigma _k} \right) \right\} \\&+(1-\eta _{ij}) (1-\eta _{ik}) \log \varPhi _2\left( \frac{u_{j}-\mu _j}{\sigma _j}, \frac{u_{k}-\mu _k}{\sigma _k}, \rho _{jk}\right) , \end{aligned}$$

where

$$\begin{aligned} {\tilde{\mu }}_j =&\mu _j + \frac{\sigma _j}{\sigma _k}\rho _{jk}(x^{(i)}_k - \mu _k), \quad {\tilde{\sigma }}_j = \sigma _j \sqrt{1-\rho _{jk}^2}. \end{aligned}$$

If $u_{j} = -\infty$, this yields a bivariate random vector with only the first variable being censored. Then the joint log-likelihood becomes

$$\begin{aligned} \ell _2^{(i)} (\rho _{jk}; \mu _j, \mu _k, \sigma _j^2, \sigma _k^2) =&\eta _{ik} \log \text {P}(Y_j = x^{(i)}_j, Y_k = x^{(i)}_k)\\&+ (1-\eta _{ik}) \log \text {P}(Y_j = x^{(i)}_j, Y_k < u_{k})\\ =&\eta _{ik}\log \left\{ \frac{1}{{\tilde{\sigma }}_k} \phi \left( \frac{x^{(i)}_k - {\tilde{\mu }}_k}{{\tilde{\sigma }}_k} \right) \frac{1}{\sigma _j} \phi \left( \frac{x^{(i)}_j-\mu _j}{\sigma _j} \right) \right\} \\&+ (1-\eta _{ik})\log \left\{ \varPhi \left( \frac{u_{k} - {\tilde{\mu }}_k}{{\tilde{\sigma }}_k} \right) \frac{1}{\sigma _j} \phi \left( \frac{x^{(i)}_j-\mu _j}{\sigma _j} \right) \right\} . \end{aligned}$$

We can solve for $\rho _{jk}$ as

4 $$\begin{aligned} {\hat{\rho }}_{jk} = \mathop {\hbox {arg max}}\limits _{\rho \in (- 1, 1)} \frac{1}{n} \sum _{i=1}^n \ell _h^{(i)}(\rho ; {\hat{\mu }}_j, {\hat{\mu }}_k, {\hat{\sigma }}_j^2, {\hat{\sigma }}_k^2), \quad h=1,2. \end{aligned}$$

Because entries in ${\hat{\varSigma }}$ are estimated separately, ${\hat{\varSigma }}$ is not guaranteed to be positive semi-definite, which is unsatisfactory because ideally we expect the empirical covariance matrix to be positive semi-definite. One way of bypassing this issue is to use the projection of ${\hat{\varSigma }}$ onto a positive semi-definite cone, as done in Fan et al. [32]. In practice, one can calculate the eigen-decomposition of ${\hat{\varSigma }}$ and threshold the negative ones to zero, which yields a new estimator ${\widetilde{ \varSigma }}$.

Given ${\widetilde{\varSigma }}$, one can apply the graphical lasso algorithm [18]

5 $$\begin{aligned} {\widetilde{\varOmega }} = \mathop {\hbox {arg max}}\limits _{\varOmega }\left\{ \log \det (\varOmega ) - \text{tr}({\widetilde{\varSigma }}\varOmega ) - \lambda _n \sum _{1\le j<k\le p}|\varOmega _{jk}| \right\} , \end{aligned}$$

to solve for the inverse covariance matrix $\varOmega$.

#### Remark 1

The graphical lasso in (5) can be replaced with other algorithms for inverse covariance matrix estimation such as the method by Cai et al. [33] or its adaptive version [34].

metaMint has been implemented in R. In particular, the optimization in (4) is solved using the optim function in R, and (5) is solved by the graphical lasso algorithm in the glasso package.

### Tuning Parameter Selection

As with other penalization-based methods, the proposed algorithm requires the specification of a tuning parameter $\lambda _n$ that controls the sparsity of the inverse covariance matrix. One can use the cross validation procedure in Guo et al. [28] or the stability approach in Liu et al. [35] to select the optimal parameter. In simulations where the ground truth is known, model selection can also be done by maximizing the accuracy in network structural recovery. In Sect. 4 on real data analysis, we used the stability approach in Liu et al. [35].

### The Modified Centered Log-Ratio

The centered log-ratio transformation is often used to transform observed microbial counts to values that are comparable across samples before downstream analysis [36–38]. Let $g({\varvec{r}}) = (\Pi _{j=1}^p r_j)^{1/p}$ denote the geometric mean of ${\varvec{r}}=(r_1,\ldots ,r_p)$. The clr of ${\varvec{r}}$ is defined as

$$\begin{aligned} \text{clr}({\varvec{r}}) = \left( \log \frac{r_1}{g({\varvec{r}})}, \ldots , \log \frac{r_p}{g({\varvec{r}})}\right) ^\intercal . \end{aligned}$$

In practice, each sample may consist of many rare species that have zero counts. Thus a pseudo count of 0.5 or 1 is often added to all counts before clr is applied. However, this practice may unfairly bias rare species and impact the accuracy in correlation estimation. The modified centered log-ratio (mclr) [22] attempts to address this limitation by transforming the non-zero counts with the usual clr and shifting all transformed values to be strictly positive.

**Fig. 2:**
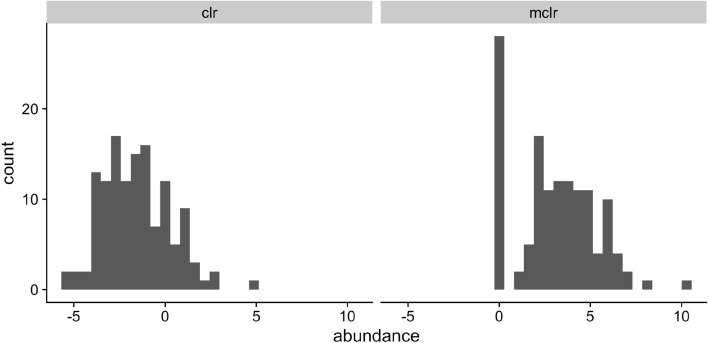
Marginal distributions of the Fusobacterium genus after the clr (left) and mclr (right) transformation to the 131 observations used in McMillan et al. [9]. A pseudo count of 0.5 was added to all counts in order to apply the clr transformation, whereas $c=0.1$ for mclr

Without loss of generality, let ${\varvec{r}}^{(i)}= ({\varvec{r}}^{(i)}_1,\mathbf{0})^\intercal = (N_i {\varvec{w}}^{(i)}_1,\mathbf{0})^\intercal$ where only components in ${\varvec{r}}^{(i)}_1$ (and ${\varvec{w}}^{(i)}_1$) are positive. Although the sample-specific scaling factor $N_i$ does not affect the relative abundances in sample *i*, it captures the variation among total sequencing reads. For example, Vandeputte et al. [39] observed up to tenfold differences in the total microbial loads after correcting for microbial cell counts. We define ${\text{mclr}}_{\varepsilon }({\varvec{r}}^{(i)})$ as $(\text{clr}({\varvec{r}}^{(i)}_1)+\varepsilon , \mathbf{0})^\intercal$, where the constant $\varepsilon$ is set to be $|\min _{i,j} \log \{r_j^{(i)}/g({\varvec{r}}_1^{(i)}\}|+c$ and $c>0$ is a small constant used to differentiate small positive counts from observed zeros. The resulting ${\text{mclr}}_{\varepsilon }({\varvec{r}}^{(i)})$ is independent of the scaling factor $N_i$, because $\text{clr}({\varvec{r}}^{(i)}_1)=\text{clr}({\varvec{w}}^{(i)}_1)$. However, adding a pseudo count to zeros and applying clr may introduce unnecessary bias towards zero counts. Figure 2 illustrates the marginal distributions of the genus *Fusobacterium* after the two transformations. Compared to clr, the mclr preserves the relative ranking of all counts while adjusting for the total sequencing depths.

Lastly, it is worth mentioning that mclr defined above is equivalent to transforming the relative abundances as done in Yoon et al. [22]. To see this, let the relative abundance ${\varvec{z}}^{(i)}$ be defined such that $z_{j}^{(i)} = r_{j}^{(i)}/S$, where $S=\sum _{j=1}^p r_{j}^{(i)}$. Moreover, we can write ${\varvec{z}}^{(i)}=({\varvec{z}}^{(i)}_1,\mathbf{0})^\intercal$ such that only components in ${\varvec{z}}^{(i)}_1$ are positive. For any $z_{j}^{(i)}>0$,

$$\begin{aligned} \log \frac{z_{j}^{(i)}}{g({\varvec{z}}_1^{(i)} )} =\log \frac{r_{j}^{(i)}}{S} - \{ \log {g({\varvec{r}}_1^{(i)})} - \log S\}= \log \frac{r_{j}^{(i)}}{g({\varvec{r}}_1^{(i)})}. \end{aligned}$$

In other words, mclr is scale invariant.

## Simulation Studies

### Model Setup

**Fig. 3:**
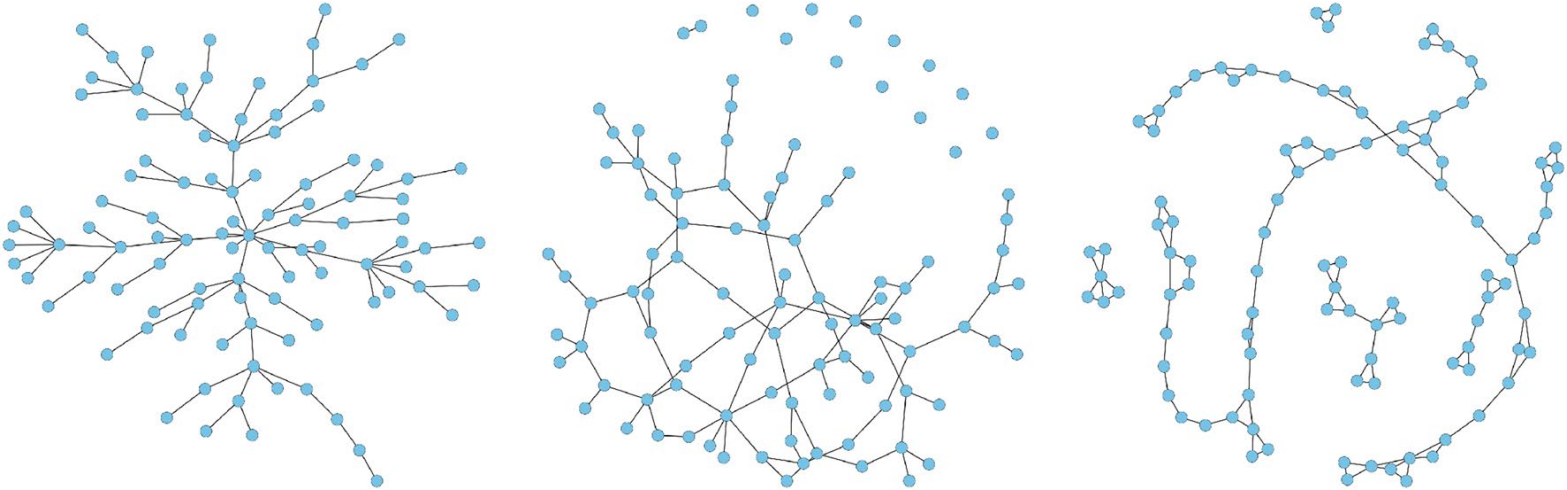
Illustration of graphs used in our simulations ($p=100$): scale-free graph (left), random graph (middle), and nearest-neighbor graph (right)

We first generated ${\varvec{y}}^{(i)}\ (i=1,\ldots ,n)$ from a multivariate normal distribution with mean ${\varvec{\mu }}_0$ and inverse covariance $\varOmega _0$. The mean parameter ${\varvec{\mu }}_0$ was generated uniformly from $[-0.5,2]$ to reflect the heterogeneity in abundances of microbial sequences and metabolites. To generate the inverse covariance matrix $\varOmega _0$, we considered the following network models, each with *p* nodes:

1. Scale-free network. This network was generated using the Barabasi-Albert algorithm [40] and has $(p-1)$ edges. The left panel of Fig. 3 illustrates a scale-free network.
2. Erdős-Rényi random graph [41]. This network has *p* edges, as illustrated in the middle panel of Fig. 3.
3. Nearest-neighbor network. We constructed this network using the same procedure described in Guo et al. [28], where we uniformly sampled *p* points on a unit square and linked any two points that are 5 nearest neighbors of each other in terms of their Euclidean distances. This network has about 2.5*p* edges. The right panel of Fig. 3 illustrates one realization of a sparse network generated with 2 nearest neighbors.

Given the network topology, the off-diagonal entries in $\varOmega _0$ were generated uniformly from $[-1,-0.5] \cup [0.5,1]$, with diagonal entries being $|\varLambda _{\min }(\varOmega _0^{-})| + 0.1$. Here $\varOmega _0^{-}$ represents the matrix $\varOmega _0$ with zeros in the diagonal and $\varLambda _{\min }(A)$ denotes the smallest eigenvalue of *A*. The covariance matrix $\varSigma _0$ is then determined by

$$\begin{aligned} \varSigma _{0,jk} = (\varOmega _0)^{-1}_{jk} /\sqrt{(\varOmega _0)^{-1}_{jj} (\varOmega _0)^{-1}_{kk}}. \end{aligned}$$

By construction, the diagonal entries of $\varSigma _0$ are all 1.

Given the latent ${\varvec{y}}^{(i)}$, the basis vector ${\varvec{w}}^{(i)} = (w_{1}^{(i)}, \ldots , w_{p}^{(i)})^{\intercal }$ was obtained through the transformation $w_{j}^{(i)} = e^{y_{j}^{(i)}}$. Censored abundances ${\varvec{r}}^{(i)} = (r_{1}^{(i)}, \ldots , r_{p}^{(i)})^{\intercal }$ were generated such that

$$\begin{aligned} r_{j}^{(i)}= {\left\{ \begin{array}{ll} N_i w_{j}^{(i)} {\varvec{I}}(y_{j}^{(i)}>0) &{} j=1,\ldots ,q,\\ w_{j}^{(i)} &{} j = q+1,\ldots ,p, \end{array}\right. } \end{aligned}$$

where $N_i$ is generated uniformly between 1 and 10. Here *q* indicates the number of microbes. Only microbiome data are assumed to be censored and compositional in this article, but this assumption can be relaxed in general. In all simulations, we set the constant $c=0.1$ in the modified clr transformation. Denote ${\varvec{x}}^{(i)}_1 = {\text{mclr}}_{\varepsilon }({\varvec{r}}_{1:q}^{(i)})$ and the observed abundances ${\varvec{x}}^{(i)}= ({\varvec{x}}^{(i)}_1,\log r^{(i)}_{q+1}, \ldots , \log r^{(i)}_{p})^\intercal$.

### Results

We compared metaMint with SPIEC-EASI [17] and gCoda [19]. The oracle estimator obtained from the latent basis $\{{\varvec{w}}^{(i)}\}_{i=1}^n$ is used as a benchmark, though in practice the oracle is generally unknown. To evaluate the performance of network recovery, we used the receiver operating characteristic (ROC) curve to plot the false positive rate (FPR) against the true positive rate (TPR) defined, respectively, as,

$$\begin{aligned} {\text{FPR}}=\frac{\sum _{1\le j<k\le p}{\varvec{I}}{(\varOmega _{0,jk}= 0, {\hat{\varOmega }}_{jk} \ne 0)}}{\sum _{1\le j<k\le p}{\varvec{I}}{(\varOmega _{0,jk}= 0)}}, \quad {\text{TPR}} =\frac{\sum _{1\le j<k\le p}{\varvec{I}}{(\varOmega _{0,jk} \ne 0, {\hat{\varOmega }}_{jk} \ne 0)}}{\sum _{1\le j<k\le p}{\varvec{I}}{(\varOmega _{0,jk} \ne 0)}}, \end{aligned}$$

where ${\hat{\varOmega }}$ denotes the estimated network. The *F*1 score [42], which is between 0 and 1, measures the accuracy of an estimator by summarizing both false positives and false negatives. Larger *F*1 scores indicate better structural recovery. For ${\hat{\varOmega }}_{\lambda ^*}$ estimated at the optimal penalty parameter ${\lambda ^*}$ selected by maximizing the *F*1 score, we also compared the entropy loss (EL) and Frobenius norm loss (FL) for estimation accuracy:

$$\begin{aligned} {\text{EL}}={\text{tr}}{(\varSigma _0{\hat{\varOmega }}_{\lambda ^*})} - \log \det (\varSigma _0{\hat{\varOmega }}_{\lambda ^*}) - p, \quad {\text{FL}} = \frac{\sum _{1\le j<k\le p}(\varOmega _{0,jk}- {\hat{\varOmega }}_{jk,{\lambda ^*}})^2}{\sum _{1\le j<k\le p}(\varOmega _{0,jk})^2}. \end{aligned}$$

**Fig. 4:**
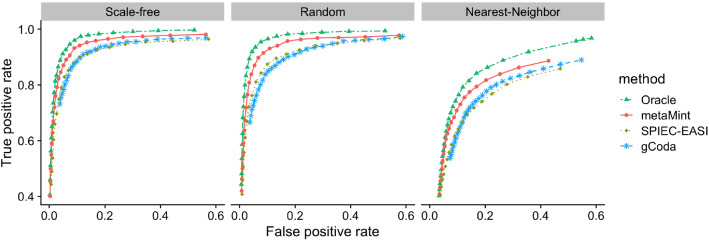
ROC curves for the first study with $p=q=60$ and $n=100$: Oracle (two-dash line in green), metaMint (solid line in red), SPIEC-EASI (dotted line in brown), and gCoda (dashed line in blue). These results are averaged over 20 replications. metaMint outperforms SPIEC-EASI and gCoda

Our first comparison is based on only microbiome data where $p=q=60$ and $n=100$. In this example, the percentage of zeros per species ranges from 0% to 70%. Input for gCoda is the censored abundance matrix ${\mathcal{D}} = ({\varvec{r}}^{(1)}+0.5, \ldots , {\varvec{r}}^{(n)}+0.5)^\intercal$. The clr transformation is then applied to each row in ${\mathcal{D}}$ and the resulting matrix is used as input for SPIEC-EASI. Figure 4 shows the ROC curves obtained from different methods across different network models. One can see that SPIEC-EASI and gCoda perform similarly, and both underperform compared to metaMint. Because the nearest-neighbor network is denser, the ROC curves in the right panel of Fig. 4 are generally lower compared to their counterparts in other network models.

**Fig. 5:**
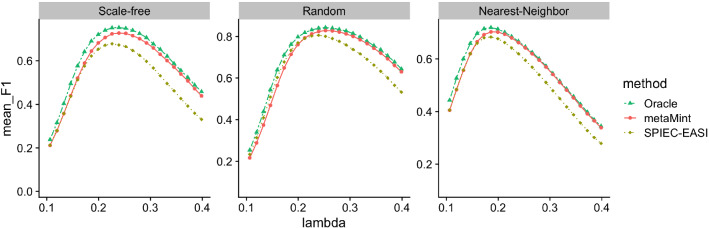
Average *F*1 scores for different methods across different network models over 50 replications in the second simulation study: Oracle (two-dash line in green), metaMint (solid line in red), and SPIEC-EASI (dotted line in brown). gCoda is not applicable in this case because it was specific for microbiome data. metaMint outperforms SPIEC-EASI

In our second study, we look at larger datasets where the number of metabolites is $q=100$ and the number of microbes is $p-q=100$. The sample size is $n=300$. The method gCoda is thus not applicable because it was proposed specifically for microbiome data. Because we only censor microbiome data, the proportion of censored variables in this example is smaller. We first compare different methods in terms of network structural recovery. Figure 5 shows the average *F*1 score of each method across a range of penalty parameters. It can be seen that metaMint has overall higher $F_1$ scores than SPIEC-EASI, and closely resembles the oracle estimator.

**Fig. 6:**
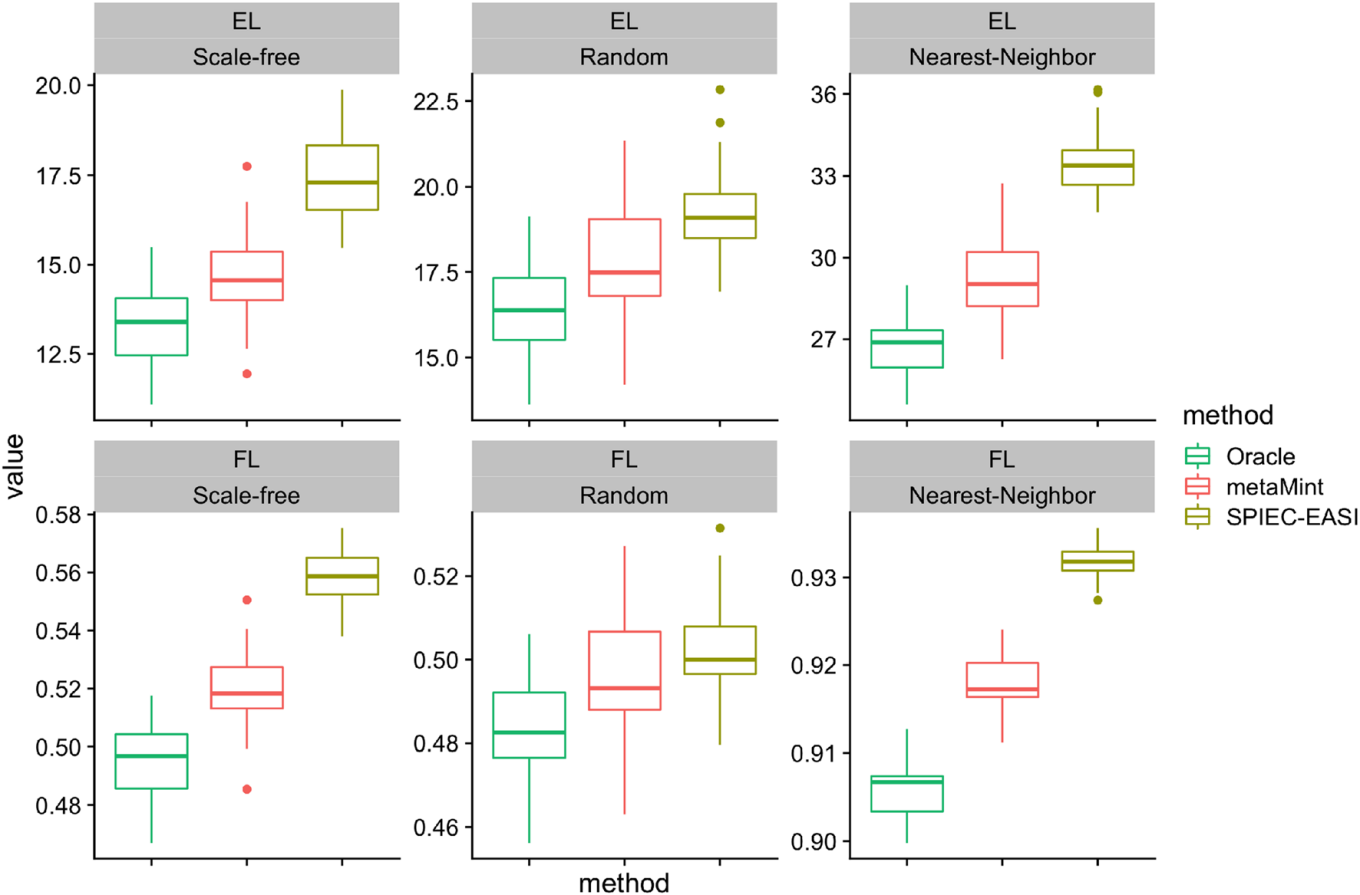
Boxplots showing the entropy loss (EL, top row) and Frobenius norm loss (FL, bottom row) for different methods across different network models over 50 replications in the second simulation study. For each method, the optimal penalty parameter was selected as the one that maximizes *F*1 score. The oracle estimator performs the best, followed by metaMint, and SPIEC-EASI has the largest entropy and Frobenius norm loss

Since we know the true network structure, we also look at comparisons in terms of inverse covariance estimation accuracy at the optimal penalty parameter selected by maximizing the *F*1 score. As shown in Fig. 6, SPIEC-EASI performs the worst in all cases because its entropy and Frobenius norm loss are the largest. It is worth pointing out that there still exists substantial gap in both EL and FL between metaMint and the oracle estimator as a result of censoring. We anticipate that this issue can be partly addressed with increased sequencing depths.

## Analysis of Bacterial Vaginosis Data

### Data Description and Processing

Bacterial vaginosis (BV) is a common vaginal condition characterized by depletion of specific Lactobacillus species and increased abundance of diverse anaerobic bacteria such as genus *Gardnerella, Prevotella and others* [43, 44]. This condition affects an estimated 30% of women at any given time [45], and is associated with increased transmission of HIV and increased risk of preterm labor [46, 47]. Improved diagnosis and treatment of BV require not only a clearer understanding of the roles of BV associated bacterial species and their interactions, but also a detailed catalog of the interactions between these bacteria and relevant metabolites. We applied the proposed multi-omic approach to a cohort of 131 Rwandan women from McMillan et al. [9]. The microbiome data from sequencing the 16S rRNA gene consist of 51 bacterial species after initial filtering, and the vaginal metabolome determined by GC-MS contains 128 metabolites [see the Methods section in 9]. One bacterial species is present in only 13 individuals, so we removed this rare species and used 50 taxa in all analysis. Of the 131 women, 79 were normal, 23 were diagnosed with BV, 22 as being intermediate between BV and the normal state, and 7 did not have diagnosis. To account for the different sequencing depths, we applied the clr and modified clr to the microbiome data. Metabolomic data available from McMillan et al. [9] have already been log transformed. After the mclr transformation, a species is treated as censored at zero if it has at least one zero count. Based on this criterion, 27 of the 50 species are left censored.

We compare metaMint with SPIEC-EASI by applying the former to mclr transformed data and the latter to clr transformed data. At the optimal tuning parameter, which was selected using the stability approach in Liu et al. [35] with pre-specified stability threshold $\alpha$, we randomly subsampled 80% of all samples to estimate the network using each method. This procedure was repeated 50 times and an edge selection frequency matrix was constructed such that each entry represents the proportion of times the corresponding edge was present. Only edges with at least 95% selection frequency were kept.

### Results

**Fig. 7:**
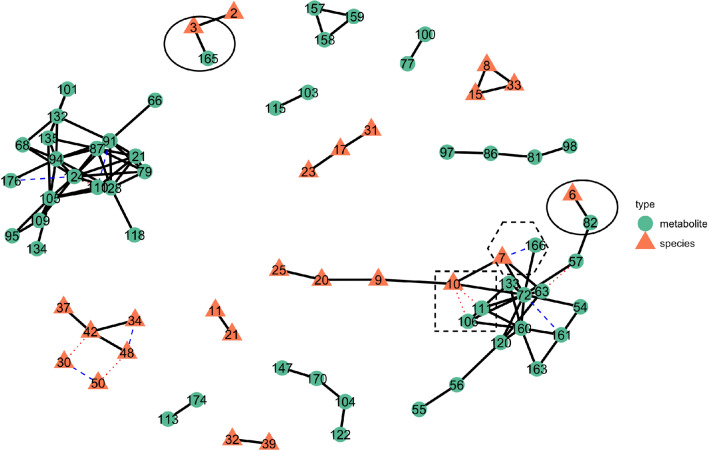
The overlayed microbe-metabolite network in the BV data example estimated from metaMint and SPIEC-EASI. Color and shape of each node indicate whether the node is a metabolite or bacterial species. Thick black edges are shared between the two methods, whereas dashed blue edges are unique to metaMint and dotted red edges are unique to the SPIEC-EASI

We first compare metaMint and SPIEC-EASI by estimating a single integrated microbe and metabolite network for all subjects at stability threshold $\alpha =0.01$. Figure 7 presents the joint microbe-metabolite network estimated by the two methods, where the thick black edges are shared between the two methods, blue edges are unique to metaMint, and red edges are unique to SPIEC-EASI. We can see that a majority of edges are shared between the two methods. In particular, both methods reported the conditional association between the genus *Gardnerella* and metabolite *GHB* (6–82), and between *Lactobacillus* and *unknown sugar 1* (3–165). These two edges are relatively stable and show up in the network for any stability threshold $\alpha \ge 0.004$. Importantly, the interaction between *Gardnerella* and *GHB* was also observed and reproduced experimentally in McMillan et al. [9]. Other notable microbe-metabolite interactions that are unique to each method include *Prevotella*—*unknown sugar 2* (7–166) estimated only by metaMint, and *Dialister*—*n-acetyl-putrescine* (10–106), *Dialister*—*phenylethylamine* (10–111) estimated only by SPIEC-EASI. These microbe-metabolite interactions are unique to each method until the stability threshold increases to $\alpha =0.02$. The differences reported by the two methods are manifestations of the different transformations and whether the model directly accounts for zero inflation.

To gain further insights into the roles of these microbe-metabolite interactions, we partitioned all subjects into two groups: the normal group ($n_1=79$) and everyone else ( the BV group, $n_2=52$). metaMint and SPIEC-EASI were applied to estimate a network for each group using the same model selection procedure as before. In general, we observe more interactions in the group-specific network estimated by SPIEC-EASI compared to the corresponding network estimated by metaMint. At stability threshold 0.01, no interaction between microbes and metabolites was recovered due to the reduced sample size in each group. As we gradually increase the stability threshold, the first pair of microbe-metabolite interaction unique to the BV group is between *Gardnerella* and *GHB*, and was identified by both metaMint and SPIEC-EASI. Table 1 provides a list of microbe-metabolite interactions that are unique to each group of patients identified by both methods at stability threshold 0.02. It is worth noting that *Gardnerella*—*GHB*, *Prevotella*—*unknown sugar 2*, and *Dialister*—*cadaverine* only show up for the BV group, whereas the interactions between *Lactobacillus* species and several metabolites appear only for the normal group. Abundance of *Lactobacillus* and *Prevotella* has long been used as a diagnostic signature for bacterial vaginosis [43, 44]. In addition, McMillan et al. [9] hypothesized that *Dialister* is responsible for malodor in the vagina. Our analysis may shed light on the mechanistic link between metabolic end products and microbes in vaginal bacterial communities, and provide key guidance regarding the diagnosis and treatment of BV.

**Table 1:** Microbe-metabolite interactions estimated by metaMint and SPIEC-EASI that are unique to each group

Microbe id	Metabolite id	Microbe name	Metabolite name	Group
1	62	Lactobacillus_iners	2-O-Glycerol-d-galactopyranoside 3	Normal
2	88	Lactobacillus_crispatus	Glyceric_acid	Normal
2	125	Lactobacillus_crispatus	Succinate	Normal
3	96	Lactobacillus	Malate	Normal
3	125	Lactobacillus	Succinate	Normal
6	82	Gardnerella	GHB	BV
7	166	Prevotella	Unknown sugar 2	BV
10	72	Dialister	Cadaverine	BV

## Discussion

The uneven sequencing depths and sparsity in microbiome data present significant challenges in inferring interactions between microbial species and their products. The different sequencing depths imply different levels of uncertainty, but how to handle varying sequencing depths in multivariate statistical analysis remains an unsolved problem [48, 49]. This paper proposes the censored Gaussian graphical model for joint estimation of microbiome and metabolomic network, which can be used to identify conditional dependencies (direct interactions) between microbial species and metabolites. Key to our proposal is the use of the modified centered log-ratio for transforming the observed microbial counts, which is scale invariant and preserves the ranking of positive counts relative to zeros. Observed zeros are attributed to undersampling and modeled as due to left censoring. Our method metaMint can be generalized to study other omics data types that fit in the censored Gaussian graphical model framework. Analysis of the bacterial vaginosis data demonstrates that metaMint facilitates the discovery of important microbe-metabolite interactions for diagnosis and treatment of this condition. The data example in Sect. 4 has about 50% censored variables, although 11 of them have less than 10% zero counts. As we move into high-resolution studies which collect microbiome data at the strain or amplicon sequence variant level, our model that explicitly accounts for observed zeros may exhibit more advantage over existing methods.

From a methodological perspective, metaMint estimates the correlations in a marginal manner, which may not be optimal because marginal approaches ignore the fact that the correlation matrix is positive semi-definite. Augugliaro et al. [26] proposed an approximated EM algorithm that jointly estimates all entries in the correlation matrix; however, their method only works well under specific settings and there is a lack of theoretical understanding about the resulting estimator. Obvious but non-trivial extension is to explore computationally and statistically efficient alternatives that jointly estimate all entries in the correlation matrix.

Our model is related to but substantially different from the zero-inflated Gaussian graphical model in McDavid et al. [27]. While our model assumes the observed zeros are due to undersampling, McDavid et al. [27] uses a two-part Hurdle model that treats all zeros as structural. The multivariate Hurdle model consists of an Ising model that captures the discrete part and a Gaussian graphical model that describes the continuous part if the hurdle is passed. When the study design favors the two-part process, as is the case in single-cell RNA-seq analysis, the multivariate Hurdle model should be considered. On the other hand, the censored Gaussian graphical model is simpler and works well if the study design favors sampling zeros and/or structural zeros can be reasonably approximated as sampling zeros [21].

It is worth pointing out that the observed data defined in (1) are continuous-valued. In this paper, we have made the simplifying assumption that the observed counts can be approximated by a log-normal distribution with left censoring. An alternative approach is to analyze observed counts directly while still treating zeros as due to left censoring. In the regression setting, Clark et al. [50] provided a general framework that uses a latent continuous variable to model observed species abundance, which can be presence/absence, continuous abundance, ordinal counts, or counts that are subject to a total sum constraint. It would be interesting to see if similar ideas can be used to model interactions between microbial species and other molecules.

## References

[1] Huttenhower C, Gevers D, Knight R, Abubucker S, Badger JH, Chinwalla AT, Creasy HH, Earl AM, FitzGerald MG, Fulton RS (2012). Structure, function and diversity of the healthy human microbiome. Nature.

[2] Lloyd-Price J, Mahurkar A, Rahnavard G, Crabtree J, Orvis J, Hall AB, Brady A, Creasy HH, McCracken C, Giglio MG (2017). Strains, functions and dynamics in the expanded human microbiome project. Nature.

[3] Callahan BJ, McMurdie PJ, Holmes SP (2017). Exact sequence variants should replace operational taxonomic units in marker-gene data analysis. ISME J.

[4] Gilbert JA, Blaser MJ, Caporaso JG, Jansson JK, Lynch SV, Knight R (2018). Current understanding of the human microbiome. Nat Med.

[5] iHMP Research Network Consortium (2019) The integrative human microbiome project. Nature 569:641–648 10.1038/s41586-019-1238-8 PMC6784865 31142853

[6] McHardy IH, Goudarzi M, Tong M, Ruegger PM, Schwager E, Weger JR, Graeber TG, Sonnenburg JL, Horvath S, Huttenhower C (2013). Integrative analysis of the microbiome and metabolome of the human intestinal mucosal surface reveals exquisite inter-relationships. Microbiome.

[7] Wu GD, Compher C, Chen EZ, Smith SA, Shah RD, Bittinger K, Chehoud C, Albenberg LG, Nessel L, Gilroy E (2016). Comparative metabolomics in vegans and omnivores reveal constraints on diet-dependent gut microbiota metabolite production. Gut.

[8] Jia W, Xie G, Jia W (2018). Bile acid-microbiota crosstalk in gastrointestinal inflammation and carcinogenesis. Nat Rev Gastroenterol Hepatol.

[9] McMillan A, Rulisa S, Sumarah M, Macklaim JM, Renaud J, Bisanz JE, Gloor GB, Reid G (2015). A multi-platform metabolomics approach identifies highly specific biomarkers of bacterial diversity in the vagina of pregnant and non-pregnant women. Sci Rep.

[10] Org E, Blum Y, Kasela S, Mehrabian M, Kuusisto J, Kangas AJ, Soininen P, Wang Z, Ala-Korpela M, Hazen SL (2017). Relationships between gut microbiota, plasma metabolites, and metabolic syndrome traits in the metsim cohort. Genome Biol.

[11] Liu R, Hong J, Xu X, Feng Q, Zhang D, Gu Y, Shi J, Zhao S, Liu W, Wang X (2017). Gut microbiome and serum metabolome alterations in obesity and after weight-loss intervention. Nat Med.

[12] Lloyd-Price J, Arze C, Ananthakrishnan AN, Schirmer M, Avila-Pacheco J, Poon TW, Andrews E, Ajami NJ, Bonham KS, Brislawn CJ (2019). Multi-omics of the gut microbial ecosystem in inflammatory bowel diseases. Nature.

[13] Gould AL, Zhang V, Lamberti L, Jones EW, Obadia B, Gavryushkin A, Korasidis N, Carlson JM, Beerenwinkel N, Ludington WB (2018). High-dimensional microbiome interactions shape host fitness. Proc Natl Acad Sci.

[14] Friedman J, Alm EJ (2012). Inferring correlation networks from genomic survey data. PLoS Comput Biol.

[15] Fang H, Huang C, Zhao H, Deng M (2015). CCLasso: correlation inference for compositional data through lasso. Bioinformatics.

[16] de la Fuente A, Bing N, Hoeschele I, Mendes P (2004). Discovery of meaningful associations in genomic data using partial correlation coefficients. Bioinformatics.

[17] Kurtz ZD, Müller CL, Miraldi ER, Littman DR, Blaser MJ, Bonneau RA (2015). Sparse and compositionally robust inference of microbial ecological networks. PLoS Comput Biol.

[18] Friedman JH, Hastie TJ, Tibshirani RJ (2008). Sparse inverse covariance estimation with the graphical lasso. Biostatistics.

[19] Fang H, Huang C, Zhao H, Deng M (2017). gCoda: conditional dependence network inference for compositional data. J Comput Biol.

[20] Kaul A, Mandal S, Davidov O, Peddada SD (2017). Analysis of microbiome data in the presence of excess zeros. Front Microbiol.

[21] Silverman JD, Roche K, Mukherjee S, David LA (2018) Naught all zeros in sequence count data are the same. bioRxiv, p 477794 10.1016/j.csbj.2020.09.014 PMC7568192 33101615

[22] Yoon G, Gaynanova I, Müller CL (2019). Microbial networks in SPRING-semi-parametric rank-based correlation and partial correlation estimation for quantitative microbiome data. Front Genet.

[23] Hoffman HJ, Johnson RE (2015). Pseudo-likelihood estimation of multivariate normal parameters in the presence of left-censored data. J Agric Biol Environ Stat.

[24] Pesonen M, Pesonen H, Nevalainen J (2015). Covariance matrix estimation for left-censored data. Comput Stat Data Anal.

[25] Jones MP, Perry SS, Thorne PS (2015). Maximum pairwise pseudo-likelihood estimation of the covariance matrix from left-censored data. J Agric Biol Environ Stat.

[26] Augugliaro L, Abbruzzo A, Vinciotti V (2018). $$\ell _1$$-penalized censored gaussian graphical model. Biostatistics.

[27] McDavid A, Gottardo R, Simon N, Drton M (2019). Graphical models for zero-inflated single cell gene expression. Ann Appl Stat.

[28] Guo J, Levina E, Michailidis G, Zhu J (2015). Graphical models for ordinal data. J Comput Gr Stat.

[29] Suggala AS, Yang E, Ravikumar P (2017) Ordinal graphical models: a tale of two approaches. In: International conference on machine learning, pp 3260–3269

[30] Tobin J (1958). Estimation of relationships for limited dependent variables. Econom: J Econom Soc.

[31] Henningsen A (2010) Estimating censored regression models in R using the censreg package. R package vignettes

[32] Fan J, Liu H, Ning Y, Zou H (2017). High dimensional semiparametric latent graphical model for mixed data. J R Stat Soc: Ser B (Stat Methodol).

[33] Cai TT, Liu W, Luo X (2011). A constrained $$\ell _1$$ minimization approach to sparse precision matrix estimation. J Am Stat Assoc.

[34] Cai TT, Liu W, Zhou HH (2016). Estimating sparse precision matrix: optimal rates of convergence and adaptive estimation. Ann Stat.

[35] Liu H, Roeder K, Wasserman L (2010) Stability approach to regularization selection (stars) for high dimensional graphical models. In: Advances in neural information processing systems, pp 1432–1440 PMC4138724 25152607

[36] van den Boogaart KG, Tolosana-Delgado R (2013). Analyzing compositional data with R.

[37] Gloor GB, Macklaim JM, Pawlowsky-Glahn V, Egozcue JJ (2017). Microbiome datasets are compositional: and this is not optional. Front Microbiol.

[38] Zhou W, Sailani MR, Contrepois K, Zhou Y, Ahadi S, Leopold SR, Zhang MJ, Rao V, Avina M, Mishra T (2019). Longitudinal multi-omics of host-microbe dynamics in prediabetes. Nature.

[39] Vandeputte D, Kathagen G, D’hoe K, Vieira-Silva S, Valles-Colomer M, Sabino J, Wang J, Tito RY, De Commer L, Darzi Y (2017). Quantitative microbiome profiling links gut community variation to microbial load. Nature.

[40] Barabási AL, Albert R (1999). Emergence of scaling in random networks. Science.

[41] Erdős P, Rényi A (1960). On the evolution of random graphs. Publ Math Inst Hung Acad Sci.

[42] van Rijsbergen CJ (1979). Information retrieval.

[43] Fredricks DN, Fiedler TL, Marrazzo JM (2005). Molecular identification of bacteria associated with bacterial vaginosis. N Engl J Med.

[44] Ravel J, Gajer P, Abdo Z, Schneider GM, Koenig SS, McCulle SL, Karlebach S, Gorle R, Russell J, Tacket CO (2011). Vaginal microbiome of reproductive-age women. Proc Natl Acad Sci.

[45] Koumans EH, Sternberg M, Bruce C, McQuillan G, Kendrick J, Sutton M, Markowitz LE (2007). The prevalence of bacterial vaginosis in the united states, 2001–2004; associations with symptoms, sexual behaviors, and reproductive health. Sex Transm Dis.

[46] Guerra B, Ghi T, Quarta S, Morselli-Labate AM, Lazzarotto T, Pilu G, Rizzo N (2006). Pregnancy outcome after early detection of bacterial vaginosis. Eur J Obstet Gynecol Reprod Biol.

[47] Atashili J, Poole C, Ndumbe PM, Adimora AA, Smith JS (2008). Bacterial vaginosis and hiv acquisition: a meta-analysis of published studies. AIDS.

[48] Weiss S, Xu ZZ, Peddada S, Amir A, Bittinger K, Gonzalez A, Lozupone C, Zaneveld JR, Vázquez-Baeza Y, Birmingham A (2017). Normalization and microbial differential abundance strategies depend upon data characteristics. Microbiome.

[49] McKnight DT, Huerlimann R, Bower DS, Schwarzkopf L, Alford RA, Zenger KR (2019). Methods for normalizing microbiome data: an ecological perspective. Methods Ecol Evol.

[50] Clark JS, Nemergut D, Seyednasrollah B, Turner PJ, Zhang S (2017). Generalized joint attribute modeling for biodiversity analysis: median-zero, multivariate, multifarious data. Ecol Monogr.

